# Vanillic acid as a promising intervention for metabolic syndrome: Preclinical studies

**DOI:** 10.22038/ijbms.2024.81709.17680

**Published:** 2025

**Authors:** Mahboobeh Ghasemzadeh Rahbardar, Gordon A Ferns, Majid Ghayour Mobarhan

**Affiliations:** 1 Applied Biomedical Research Center, Mashhad University of Medical Sciences, Mashhad, Iran; 2 Brighton and Sussex Medical School, Division of Medical Education, Falmer, Brighton BN1 9PH, Sussex, UK; 3 Department of Nutrition, Faculty of Medicine, Mashhad University of Medical Sciences, Mashhad, Iran; 4 Iranian UNESCO Center of Excellence for Human Nutrition, Mashhad University of Medical Sciences, Mashhad, Iran; 5 Metabolic Syndrome Research Center, Mashhad University of Medical Sciences, Mashhad, Iran

**Keywords:** Cardiovascular diseases, Diabetes mellitus type 2, Glucose, Insulin resistance, Lipid metabolism, Obesity, Phenolic acid

## Abstract

Metabolic syndrome is a clustering of metabolic abnormalities and anthropometric factors that increase the risk of cardiovascular disease and type 2 diabetes mellitus. As the search for effective treatments intensifies, attention has turned towards natural substances with potential medicinal benefits. Among them, vanillic acid, a phenolic acid present in many plants, has attracted some attention due to its wide range of biological activities. This review aimed to provide an in-depth summary of the potential therapeutic use of vanillic acid in metabolic syndrome. The potential mechanisms of action of vanillic acid, including its anti-oxidant, anti-inflammatory, and hypolipidemic properties, are discussed. The effect of vanillic acid on glucose homeostasis, insulin sensitivity, and adipocyte activity is also addressed. The effect of vanillic acid on lipid metabolism, including the control of lipid synthesis, breakdown, and transport, is also reviewed. The emerging evidence for the beneficial effects of vanillic acid in animal models, *in vitro *studies, and preliminary clinical studies is also highlighted. The data suggests that vanillic acid has the potential to ameliorate metabolic syndrome. However, further preclinical and clinical research is needed to determine the specific mechanisms of action, appropriate dose, and subsequent advantages of vanillic acid. A more comprehensive understanding of the therapeutic potential of vanillic acid could pave the way for developing innovative techniques for preventing and treating metabolic syndrome and its implications.

## Introduction

Metabolic syndrome is characterized by a clustering of metabolic and anthropometric abnormalities, including insulin resistance, dyslipidemia, hypertension, and obesity; it has become a global health concern due to its rising prevalence and association with increased cardiovascular disease and type 2 diabetes risk (1). The reported prevalence of metabolic syndrome varies greatly due to various factors, including the specific diagnostic criteria used and the demographic characteristics of the study population (including age, ethnic composition, gender, and socioeconomic status of the cohorts under investigation). These differences highlight the complexities of metabolic syndrome and the effect of many variables on its incidence. Furthermore, abdominal fat accumulation, high-calorie consumption, and a sedentary lifestyle are identified as major parameters that trigger the development of metabolic syndrome (2). The multifactorial nature of metabolic syndrome necessitates exploring novel therapeutic strategies to manage its complex pathophysiology effectively.

Natural compounds with diverse bioactive properties such as *Elettaria cardamomum* (3), *Nigella sativa* (4), *Portulaca oleracea* (1), *Solanum melongena* (5), Dendrobium (6), alpha-mangostin (7), alpha-lipoic acid (8), zeaxanthin (9), and ellagic acid (10) have attracted the interest of researchers in recent years as potential interventions for metabolic syndrome. Among these natural compounds, vanillic acid, a phenolic acid commonly found in various plant sources, has emerged as a promising candidate with apparent health benefits. 

Vanillic acid, chemically known as 4-hydroxy-3-methoxybenzoic acid, is a derivative of vanillin, the primary component responsible for vanilla bean’s characteristic flavor and aroma (11). However, vanillic acid exists naturally in a wide variety of species, including *Angelica sinensis*, benzoin, olives, and soybeans (11). It is usually seen as white or yellow powder or crystals with a pleasant odor. Vanillic acid is commonly used as a fragrance and flavoring ingredient due to its aromatic characteristics. Natural vanillic acid is present in a wide range of food sources, including guava, oranges, and rice grains (12). Beyond its traditional culinary uses, vanillic acid has attracted attention for its broad spectrum of biological activities, including anti-oxidant (13), anti-inflammatory (14), cardioprotective (15), hepatoprotective (16), anti-hyperinsulinemic (17), and anti-obesity (18) effects. These properties have raised interest in investigating the potential therapeutic role of vanillic acid in the context of metabolic syndrome.

This review aims to provide a comprehensive summary of the role of vanillic acid in metabolic syndrome, shedding light on its mechanisms of action and potential therapeutic implications. By analyzing the current body of literature, both *in vitro *and *in vivo*, we seek to elucidate the impact of vanillic acid on key components of metabolic syndrome, including insulin resistance, dyslipidemia, hypertension, and obesity. Understanding the underlying mechanisms through which vanillic acid exerts its effects on metabolic syndrome may contribute to our knowledge of disease pathogenesis and pave the way for developing novel strategies to prevent and treat this complex metabolic disorder. 

## Methods

**Figure 1:**
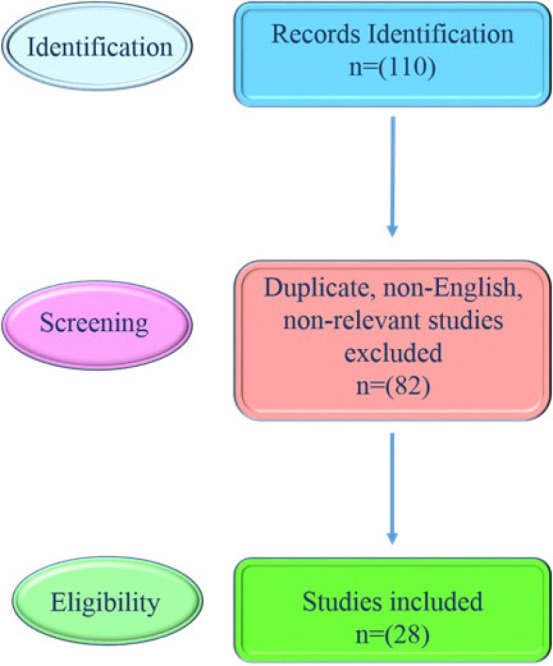
Search criteria flow chart

**The present study involved an extensive search across various databases, including Scopus, Google Scholar, and PubMed, without specific time limitations up to August 2024. The search encompassed a wide range of articles that included** ***in vitro*** **investigations,** ***in vivo*** **experiments, and clinical trials. To ensure a thorough exploration, numerous search terms were utilized, such as “vanillic acid”, “atherosclerosis”, “anti-obesity”, “anti-hypertensive”, “anti-hyperlipidemic”, “anti-hyperglycemic”, “anti-diabetic”, “blood pressure”, “blood glucose”, “dyslipidemia”, “diabetes”, “hypotensive”, “hypoglycemic”, “hypertriglyceridemia”, “high triglyceride”, “hypertension”, “hyperlipidemia”, “hyperglycemia”, “hypercholesterolemia”, “high cholesterol”, “weight loss”, “serum lipids”, “overweight”, “obesity”, “metabolic syndrome”, and “insulin”.** Figure 1 **shows the search criteria flow chart.**

**Specific inclusion and exclusion criteria were used to identify relevant published articles. Inclusion criteria included papers examining the effects of vanillic acid on metabolic disorders and associated factors, studies investigating the impact of vanillic acid across** ***in silico*** **,** ***in vitro*** **, animal models, and human subjects, and English-language, peer-reviewed journal articles providing precise academic information. Non-English articles, research not focused on metabolic disorders or the effects of vanillic acid, and non-peer-reviewed content such as editorials, letters, and conference abstracts were excluded since they lacked the rigorous evaluation required by peer review processes.**

Role of vanillic acid in managing metabolic syndrome

*Diabetes*

Diabetes and metabolic syndrome are linked by common underlying processes that contribute to their development and progression. 

Oxidative stress, which results from an imbalance between the anti-oxidant defense system of the body and the formation of reactive oxygen species (ROS), is a key factor in the development of both metabolic syndrome and diabetes. Overproduction of ROS damages cells, impairing insulin signaling and triggering insulin resistance (6, 19). Inflammation is crucial in the pathophysiology of metabolic syndrome and diabetes. Chronic low-grade inflammation, characterized by high levels of pro-inflammatory cytokines, disrupts insulin signaling pathways and increases insulin resistance. Furthermore, inflammation contributes to adipose tissue malfunction, resulting in the release of adipokines, which promote insulin resistance and metabolic abnormalities (6, 20). Another key mechanism linked to metabolic syndrome and diabetes is apoptosis. Increased apoptosis has been seen in pancreatic beta cells, which produce insulin, and other tissues affected by metabolic syndrome. This increased cell death leads to a reduction in beta cell mass and function, leading to insulin insufficiency and glucose control (21). 

The protein kinase B (Akt) and extracellular signal-regulated protein kinases 1 and 2 (ERK1/2) signaling pathways are critical regulators of insulin function and glucose metabolism. These pathways are dysregulated in metabolic syndrome and diabetes, resulting in decreased insulin signaling and reduced glucose uptake by target tissues. Akt activation is required for insulin-mediated glucose transfer, whereas Erk1/2 activation regulates insulin production and beta cell function (22-24). Furthermore, metabolic syndrome and diabetes are associated with dysregulation of nuclear factor erythroid 2–related factor 2 (Nrf2), a transcription factor involved in cellular anti-oxidant defense. Reduced Nrf2 activity decreases the production of anti-oxidant enzymes and impairs the ability to counteract oxidative stress, increasing oxidative damage and contributing to the development of both disorders (5, 25, 26).

Developing successful therapies requires understanding the complex relationship between metabolic syndrome and diabetes and the underlying processes, including oxidative stress, apoptosis, inflammation, Akt, Erk1/2 activation, and Nrf2 dysregulation. In the subsequent sections of this review, we will explore the potential of vanillic acid as a promising intervention for diabetes.

***In silico***

Several vanillic acid derivatives have been developed and examined in a study to demonstrate the potential inhibition of amylolytic enzymes. Using *in silico* docking, the link between the ligand and receptor molecule was verified. The findings showed that two derivatives (3a and 3g) had good glide scores for developing α-amylase and α-glucosidase selective inhibition (27). In another study, the binding energies of vanillic acid against α-glucosidase and α-amylase were found to be −61.63 and −65.52, respectively. Moreover, the binding energies against tumor necrosis factor-alpha (TNF-α) and interleukin (IL)-6 were -78.0683 and -62.116, respectively (28). The ability of vanillic acid to inhibit α-glucosidase and α-amylase was examined in another study. Ten Thai colored rice cultivars were examined for their phenolic chemical levels, and the association between the inhibitory effect of the colored rice extract and vanillic acid was assessed. The findings demonstrated that the inhibitory effect of vanillic acid against α-glucosidase was greater than that against α-amylase. According to docking research, vanillic acid and the amino acid at the binding sites of α-glucosidase and α-amylase exhibit strong binding by three and four hydrogen bonds, respectively. The rice cultivars with the highest concentrations of vanillic acid phenolic compounds were found in the methanol extracts (29). Moreover, another *in silico* study found that vanillic acid can bind to the active sites of the glycogen synthase kinase-3 beta complex, phosphatidylinositol 3-kinase, insulin receptor substrate 1, and protein kinase 1 (30).

*In vitro*

Vanillic acid treatment of HepG2 cells during hyperinsulinemic shock, caused by 1 μM insulin for 24 hours, protected mitochondria, enhanced cell viability, reduced lipid peroxidation, ROS, and glycation. Moreover, vanillic acid exerted its beneficial effects through the AMP-activated protein kinase (AMPK)/Sirtuin1 (Sirt1)/peroxisome proliferator-activated receptor gamma coactivator 1-alpha (PGC-1α) pathway (31). Another similar study reported that vanillic acid could safeguard glucokinase inhibition, enhance the activity of Bcl-2-associated death receptor (BAD), and decrease the reduction of glycogen synthesis in HepG2 cells during hyperinsulinemic shock (32). A recent *in vitro *investigation claimed that vanillic acid protected HepG2 cells against hyperinsulinemia through its antilipogenic, anti-oxidant, endoplasmic reticulum**,** and mitochondrial protection effects (17). It has also been shown that pretreating pancreatic β-cells with vanillic acid and then exposing them to H_2_O_2_ increased cell viability, augmented ERK1/2 activation, insulin secretion, expression of catalase, superoxide dismutase (SOD)-2, Nrf2, and Akt. It also lowered the expression of Bcl-2-associated X protein (BAX) and BAD (33).

*Research incorporating both in vitro and in vivo studies*

Treating insulin-resistant FL83B mouse hepatocytes with vanillic acid significantly increased glucose uptake. Besides, in the *in vivo* part of the research, vanillic acid enhanced the expression of hepatic insulin-signaling proteins and lowered serum insulin and glucose levels in rats with a high-fat diet (34). It has been shown that treating RAW 264.7 cells exposed to bovine serum albumin with vanillic acid increased cell viability and prevented bovine serum albumin glycation. The results of the *in vivo* part of the study illustrated that intraperitoneal injection of vanillic acid to diabetic rats had no significant effect on the amounts of serum glycated hemoglobin (HbA1c) and fructosamine (35).

*In vivo*

Vanillic acid enhanced anti-oxidant activities and attenuated fasting blood glucose (FBG), insulin, and lipid peroxidation markers in high-fat diet-induced diabetic rats (36). It has been reported that administering vanillic acid to diabetic rats resulted in increased amounts of insulin and anti-oxidant enzymes. It also reduced the levels of blood glucose, HbA1c, glucose-6-phosphatase, thiobarbituric acid reactive substance (TBARS), lipid hydroperoxide (LOOH), fructose 1,6-bisphosphatase, TNF-α, interleukin IL-1β, and IL-6 (37). In streptozotocin-induced diabetic rats, vanillic acid reduced hyperglycemia and HbA1c (38). The oral administration of vanillic acid to rats with diabetic neuropathy could significantly reduce blood glucose levels (39). Likewise, supplementation of vanillic acid to rats with diabetic nephropathy decreased hyperglycemia (40) (Table 1).

In brief, the accumulating evidence from *in vitro *and *in vivo* studies suggests that vanillic acid holds promise as a potential intervention for metabolic syndrome and diabetes. Its multiple effects on various molecular pathways, including oxidative stress, apoptosis, insulin signaling, and inflammation, make it a favorable strategy for managing metabolic syndrome’s complex and interconnected components. The precise underlying mechanisms through which vanillic acid exerts its effects on diabetes and metabolic syndrome are still being investigated. However, studies suggest that the compound may act through various pathways, including the activation of the AMPK/Sirt1/PGC-1α pathway, modulation of insulin signaling pathways such as Akt and ERK1/2, and regulation of key enzymes and proteins involved in glucose and lipid metabolism. While the findings thus far are promising, further research is needed to fully understand the therapeutic potential of vanillic acid and its optimal dosage, safety profile, and long-term effects in human subjects. Clinical trials are necessary to determine its effectiveness as a potential therapeutic intervention for individuals with diabetes and metabolic syndrome.

**Table 1:** Therapeutic potential of vanillic acid in diabetes

Study design	Doses/Duration	Results	Ref.
*in vitro*			
*In vitro,* HepG2 cells	5 and 10 μM	-Protected mitochondria ↓ Lipid peroxidation, ROS, glycation	(31)
*In vitro,* HepG2 cells	5 and 10 μM	-Protected glucokinase inhibition ↑ BAD activity ↓ Reduction of glycogen synthesis	(32)
*In vitro,* HepG2 cells		-Protected endoplasmic reticulum and mitochondrial ↑Antioxidant and antilipogenic activities	(17)
*In vitro,* pancreatic β-cells		↑ Cell viability, Erk1/2 activation, insulin secretion, expression of catalase, SOD-2, Nrf2, and Akt ↓ Expression of BAX and BAD	(33)
*in vitro *plus* in vivo*			
*In vitro,* FL83B mouse hepatocytes	12.5-100 μM, 6.25 ng/ml	↑ Glucose uptake	(34)
*In vivo*, male Sprague-Dawley rats	30 mg/kg, 3 weeks, PO	↑Expression of hepatic insulin-signaling proteins ↓Serum insulin and glucose	
*In vitro,* RAW 264.7 cells	7.8–500 μM, 48 hr	↑ Cell viability ↓ Bovine serum albumin glycation	(35)
*In vivo*, male Sprague Dawley rats	0, 1.5, 4.5, and 15 mg/kg, 4 weeks, IP	-No significant effect on the amounts of serum HbA1c and fructosamine	
*In vivo*			
*In vivo,* male Wistar rats	50 mg/kg, 8 weeks, PO	↑ Antioxidant activities ↓FBS, insulin, lipid peroxidation markers	(36)
*In vivo,* male albino Wistar rats	25 and 50 mg/kg, 45 days, PO	↑Insulin levels, antioxidant enzymes ↓ Blood glucose, HbA1c, glucose-6-phosphatase, TBARS, LOOH, fructose 1,6-bisphosphatase, TNF-a, IL-1β, and IL-6	(37)
*In vivo,* male Sprague-Dawley rats	50 and 100 mg/kg, six weeks, PO	↓ Hyperglycemia, HbA1c	(38)
*In vivo*, female Wistar albino rats	25, 50, and 100 mg/kg, four weeks, PO	↓ Blood glucose	(39)
*In vivo, *rats	25, 50, and 100 mg/kg, 6 weeks, PO	↓ Hyperglycemia	(40)

Akt: protein kinase B; BAD: Bcl-2-associated death receptor; BAX: Bcl-2-associated X protein; Erk1/2: extracellular signal-regulated protein kinases 1 and 2; FBS: fasting blood glucose; HbA1c: glycated hemoglobin; IL: interleukin; LOOH: lipid hydroperoxide; Nrf2: nuclear factor erythroid 2–related factor 2; ROS: reactive oxygen species; SOD: superoxide dismutase; TBARS: thiobarbituric acid reactive substance; TNF-α: tumor necrosis factor-alpha

**Table 2:** Therapeutic potential of vanillic acid in dyslipidemia

Study design	Doses/Duration	Results	Ref.
*In vivo*			
*In vivo*, male albino Wistar rats	5 and 10 mg/kg, 10 days, PO	↑ HDL–C ↓ Serum cholesterol, triglycerides, FFA, LDL-C, VLDL-C, HMG-CoA reductase and lecithin cholesterol acyl transferase activity	(57)
*In vivo*, male albino Wistar rats	50 mg/kg, 30 days, PO	↑ HDL–C ↓Serum cholesterol, FFA, LDL–C, VLDL–C, phospholipids, triglycerides, HMG-CoA reductase activity in the plasma	(58)
*In vivo*, male rats	50 mg/kg, 8 weeks, PO	↓ Cholesterol and triglycerides	(59)
*In vivo*, male Albino Wistar rats	1 and 3% of diet, two months, PO	↓ Triglycerides and LDL levels	(60)
*In vivo*, male Albino Wister rats	8 weeks	↑ HDL levels ↓ Lipid profile	(61)

FFA: free fatty acids; HDL–C: high-density lipoprotein-cholesterol; HMG-CoA: 3-hydroxy 3-methylglutaryl coenzyme A; LDL-C: low-density lipoprotein-cholesterol; VLDL-C: very low-density lipoprotein-cholesterol

**Figure 2:**
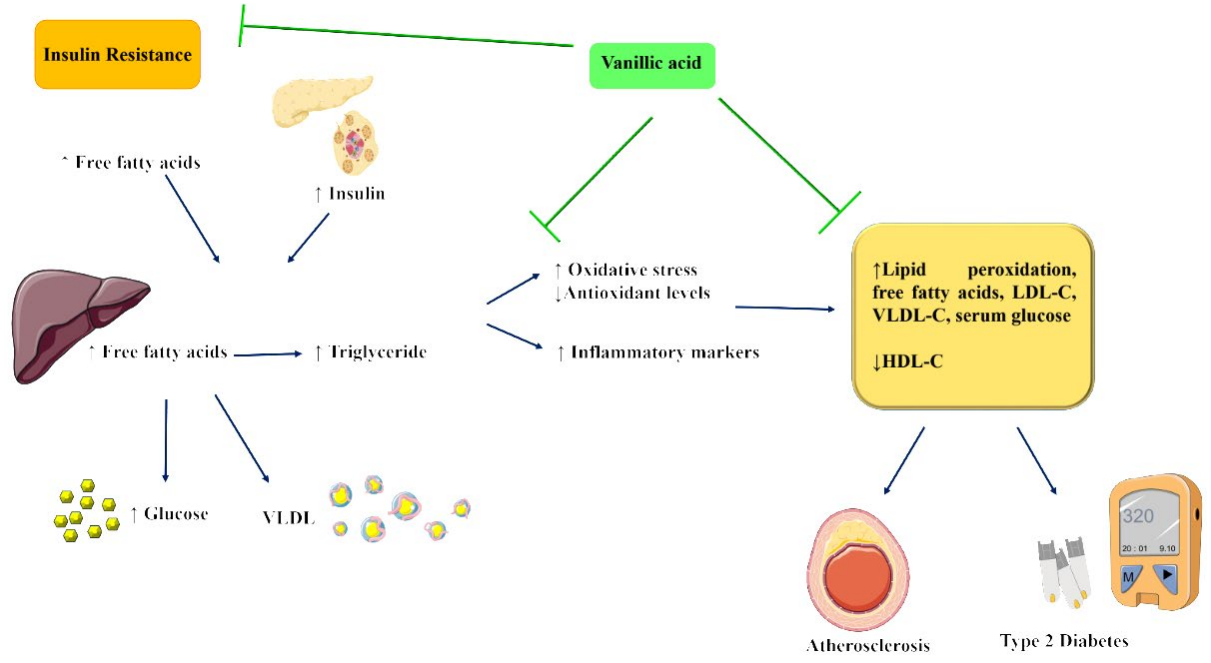
The proposed mechanism of anti-hyperlipidemic effect of vanillic acid (Images from https://smart.servier.com)

**Table 3:** Therapeutic potential of vanillic acid in obesity

Study design	Doses/Duration	Results	Ref.
*in vitro *plus* in vivo*			
*In vitro,* HepG2 hepatocytes, brown pre-Adipocytes, primary cultured brown Adipocytes, and 3T3-L1 cells	0, 0.1, 1, and 10 μM, six days	↑ Thermogenesis and mitochondria-related factors in primary cultured brown adipocytes ↓ Hepatotoxic/inflammatory markers in HepG2 hepatocytes, differentiation of 3T3-L1 adipocytes	(71)
*In vivo*, male C57BL/6J mice	10, 100, and 1000 mg/kg, six weeks, PO	↑ AMPKα in liver and white adipose tissue ↓ Body weights, PPARγ and C/EBPα in liver and white adipose tissue, lipid accumulation, hepatotoxic/inflammatory markers in liver tissue	
*In vivo*			
*In vivo*, male C57BL/6J mice	0.5% w/w, 16 weeks, PO	- Preserved body temperature ↑ Glucose tolerance, mitochondrial synthesis, and thermogenesis of inguinal white adipose tissue and brown adipose tissue ↓ Body weight gain, insulin resistance	(72)
*In vivo*, male C57BL6/J mice	10 mg/kg, 2 weeks, PO	↑ AMPK activation and thermogenesis in white adipose tissue ↓White adipose tissue weight, body weight	(73)

AMPK: AMP-activated protein kinase; C/EBPα: CCAAT/enhancer-binding protein α; PPARγ: peroxisome proliferator-activated receptor γ

**Figure 3:**
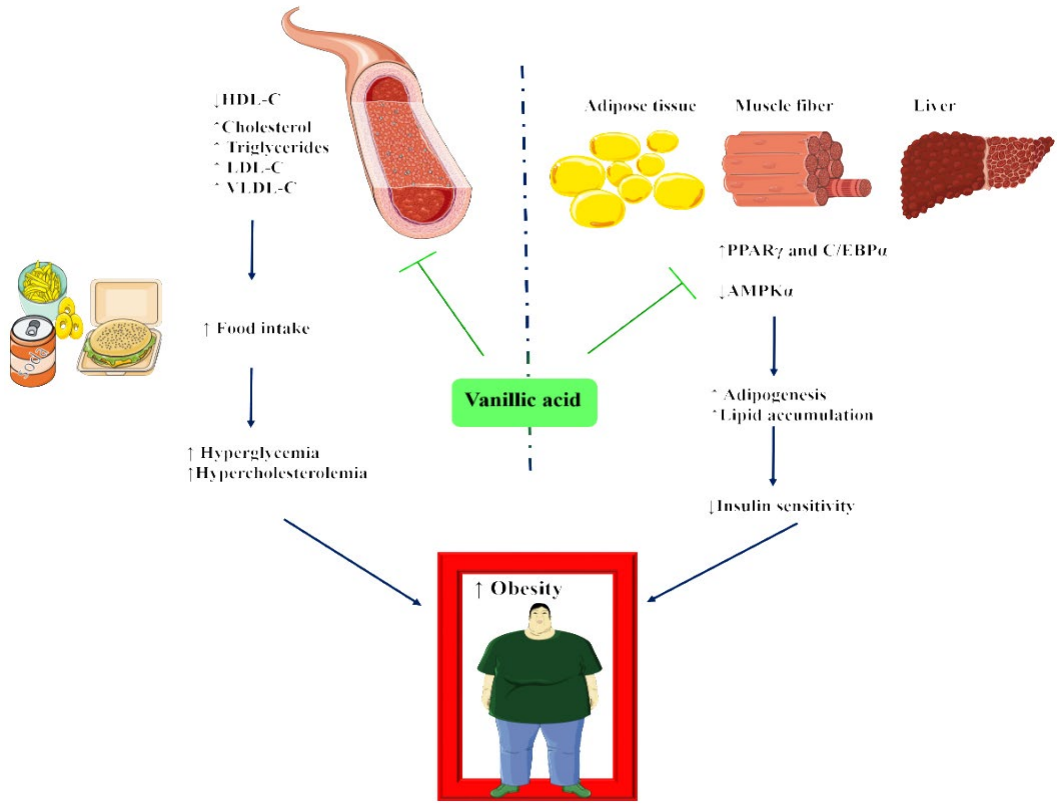
Suggested underlying mechanism of anti-obesity effect of vanillic acid (Images from https://smart.servier.com)

**Table 4:** Therapeutic potential of vanillic acid in hypertension

Study design	Doses/Duration	Results	Ref.
*In vivo*			
*In vivo*, male albino Wistar rats	25, 50, and 100 mg/kg, 30 days, IG	↑ NO metabolites concentration in plasma, SOD, CAT, GPx, vitamin C, vitamin E, and GSH ↓ Systolic and diastolic blood pressures, lipid peroxidation products	(80)
*In vivo*, male albino Wistar rats	50 mg/kg, 4 weeks, IG	-Ameliorated left ventricular function ↑ Aortic eNOS expression ↓MAP, hear rate	(81)
*In vivo*, male Wistar rats	10 mg/kg, ten days, gavage	↑ eNOS mRNA expression levels in cardiac tissue ↓ Left ventricular end-diastolic pressure, iNOS mRNA expression	(82)
*In vivo*, male Wistar rats	10 mg/kg, ten days, gavage	↑SOD, CAT, and GPx in blood ↓Blood pressure, XOX, MDA	(82)
*In vivo*, male Albino Wistar rats	10, 20, and 40 mg/kg, four weeks, PO	-Restored heart rate and systolic blood pressure	(83)
*In vivo*, male Sprague‒Dawley rats	25, 50, and 100 mg/kg, 4 weeks, IG	↑ P-eNOS/eNOS levels, NO amounts ↓ Pulmonary tissue fibrosis, protein and gene expression of Arg2, Hif-1β, and Hif-2α, right ventricular systolic pressure	(84)

Arg2: Arginase-2; CAT: catalase; eNOS: endothelial nitric oxide synthase; GPx: glutathione peroxidase; GSH: reduced glutathione; Hif: hypoxia-inducible factor; iNOS: inducible nitric oxide synthase; MAP: mean arterial pressure; MDA: malondialdehyde; mRNA: messenger ribonucleic acid; NO: nitric oxide; SOD: superoxide dismutase; XOX: xanthine oxidase

*Dyslipidemia*

Dyslipidemia is defined as elevated levels of lipids in the blood, including total cholesterol (≥200 mg/dl), low-density lipoprotein cholesterol (LDL-C ≥130 mg/dl), very low-density lipoprotein cholesterol (VLDL-C ≥30 mg/dl), triglycerides (≥150 mg/dl), and decreased levels of high-density lipoprotein cholesterol (HDL-C <40 mg/dl for men, <50 mg/dl for women), plays an important role in the development and progression of metabolic syndrome (41, 42). These lipid abnormalities lead to lipid metabolism dysregulation and increase the development of atherosclerosis and cardiovascular disorders associated with metabolic syndrome (43).

Several critical components are involved in the underlying processes that connect hyperlipidemia and metabolic syndrome. Elevated LDL-C and VLDL-C levels contribute to the accumulation of cholesterol-rich particles in the arterial walls, resulting in the development of atherosclerotic plaques (44, 45). Furthermore, low HDL-C levels impair reverse cholesterol transport, which allows the removal of excess cholesterol from peripheral tissues (46). Imbalances in lipid metabolism are frequently accompanied by alterations in the activity of enzymes involved in cholesterol synthesis, such as 3-hydroxy 3-methylglutaryl coenzyme A (HMG-CoA) reductase, which is essential in the production of cholesterol (47, 48). HMG-CoA reductase activity dysregulation can lead to high cholesterol levels in those with metabolic syndrome (49).

Furthermore, oxidative stress and inflammation are critical in the development of hyperlipidemia and metabolic syndrome (50). Increased oxidative stress can cause lipid peroxidation, damaging lipids and lipoproteins. This mechanism aggravates the progression of atherosclerosis and the development of cardiovascular complications (51). Inflammatory mechanisms also have a role in the development of hyperlipidemia in patients with metabolic syndrome (52). Inflammatory cytokines can boost acute-phase protein synthesis and impair lipid metabolism (53, 54). Furthermore, prolonged low-grade inflammation worsens insulin resistance, adding to lipid metabolic dysregulation (55, 56).

Understanding the intricate relationship between metabolic syndrome and dyslipidemia, including oxidative stress, inflammation, lipid profiles, and enzymatic activity, is key to developing effective treatment options. In this context, vanillic acid may serve as a promising therapeutic intervention for hyperlipidemia and its related implications due to its possible therapeutic effects on oxidative stress and lipid metabolism.

*In vivo*

Treating rats with vanillic acid significantly increased plasma HDL–C concentrations and decreased serum total cholesterol, triglycerides, free fatty acids (FFA), LDL–C, VLDL–C, HMG-CoA reductase, and lecithin cholesterol acyl transferase activity (57). It has been reported that the administration of vanillic acid to hypertensive rats resulted in augmented HDL–C and attenuated serum cholesterol, FFA, LDL–C, VLDL–C, phospholipids, triglycerides, as well as HMG-CoA reductase activity in the plasma (58) (Table 2). The supplementation of vanillic acid besides exercise training in rats with fatty liver and insulin resistance lowered plasma cholesterol and triglyceride levels (59). Adding sardine oil-loaded vanillic acid-grafted chitosan microparticles to rats’ diets significantly reduced plasma triglycerides and LDL levels (60). Likewise, vanillic acid was shown to decrease lipid profile except for HDL in diabetic hypertensive rats with a high-fat diet (61). 

In summary, vanillic acid has shown potentially beneficial effects in improving lipid profiles and reducing risk factors associated with cardiovascular diseases in *in vivo* studies, specifically in rat models (Figure 2). It has been observed to increase levels of HDL-C while decreasing serum cholesterol, triglycerides, FFA, LDL-C, VLDL-C, HMG-CoA reductase, and lecithin cholesterol acyl transferase activity in these animal models. More study is required to understand the underlying processes better and assess the safety and long-term consequences of supplementing with vanillic acid in human subjects. However, these investigations offer insightful information about the possible therapeutic uses of vanillic acid in treating dyslipidemia and associated disorders.

*Obesity*

Central obesity is a key feature of metabolic syndrome (1, 3). The excessive accumulation of adipose tissue in obesity leads to dysregulation of adipocyte function and the release of pro-inflammatory adipokines, contributing to insulin resistance, dyslipidemia, and hypertension (62, 63). In this field, the transcription factors peroxisome proliferator-activated receptor γ (PPARγ) and CCAAT/enhancer-binding protein α (C/EBPα) play crucial roles in adipogenesis and lipid metabolism (64, 65). Their overexpression in adipocytes promotes adipocyte differentiation and lipid storage, exacerbating obesity-related metabolic dysfunction (66, 67). Conversely, activation of AMPK, a cellular energy sensor, has been shown to counteract adipogenesis and improve metabolic parameters associated with obesity (68, 69). AMPK activation enhances energy expenditure, inhibits lipid synthesis, and promotes fatty acid oxidation (70). Therefore, targeting PPARγ, C/EBPα, and AMPK has emerged as a potential therapeutic approach to mitigate obesity and its associated metabolic syndrome. Understanding the complicated relationship between these molecular mechanisms may provide insights into developing novel interventions for treating obesity and preventing the progression of metabolic syndrome. In the following sections of this review, we will delve into the potential of vanillic acid, an optimistic intervention that holds promise for the future of obesity treatment.

*Research incorporating both in vitro and in vivo aspects*

Administration of vanillic acid significantly decreased body weight in mice on a high-fat diet and genetically obese db/db mice. Vanillic acid treatment reduced the expression of two major markers of adipogenesis, PPARγ and C/EBPα, while increasing the activity of AMPK in both white adipose tissue and liver tissue of the treated mice. Additionally, vanillic acid inhibited lipid accumulation and reduced hepatotoxicity and inflammation markers in liver tissues of the mice and HepG2 hepatocytes. Vanillic acid treatment also decreased the differentiation of 3T3-L1 adipocytes by regulating adipogenic factors, including PPARγ and C/EBPα. To further investigate the relationship between vanillic acid and AMPK, small interfering ribonucleic acid (RNA) was used to silence AMPK in 3T3-L1 cells. In these AMPK-deficient cells, the inhibitory effects of vanillic acid on PPARγ and C/EBPα were diminished. Furthermore, in brown adipose tissue of mice and primary cultured brown adipocytes, vanillic acid increased the expression of factors related to mitochondria and thermogenesis, such as uncoupling protein 1 and PPARγ-coactivator 1-α (71).

*In vivo*

By adding vanillic acid to the diet of mice with a high-fat and high-fructose diet, researchers observed several positive effects, including preserved body temperature by growing the mitochondrial synthesis of brown adipose tissue, enhanced glucose tolerance, increased mitochondrial synthesis and thermogenesis in both inguinal white adipose tissue and brown adipose tissue, and reduced body weight gain. Additionally, vanillic acid supplementation decreased insulin resistance (72) (Table 3). The administration of vanillic acid to obese mice with a high-fat diet boosted AMPK activation and thermogenesis in white adipose tissue, thereby reducing white adipose tissue and body weight (73).

Briefly, the administration of vanillic acid has demonstrated significant positive effects on obesity and metabolic disorders. Treatment with vanillic acid resulted in a decrease in body weight and inhibited the expression of two major markers of adipogenesis, PPARγ and C/EBPα. Vanillic acid treatment also showed an increase in the activity of AMPK in both white adipose tissue and liver tissue (Figure 3). This suggests that vanillic acid may exert its beneficial effects by activating the AMPK signaling pathway. Furthermore, vanillic acid exhibited promising outcomes in reducing lipid accumulation, hepatotoxicity, and inflammation markers in the liver. It also regulated the factors involved in the differentiation of adipocytes, thereby decreasing their formation. Additionally, vanillic acid showed potential in enhancing mitochondrial synthesis and promoting thermogenesis in adipose tissues, resulting in improved glucose tolerance, reduced body weight gain, and decreased insulin resistance. These results demonstrate vanillic acid’s potential as a medicinal product that targets important adipogenic factors**,** including C/EBPα and PPARγ, to treat obesity and related metabolic disorders. In order to comprehend the underlying molecular pathways and investigate their possible therapeutic uses, more study is required.

*Hypertension*

Hypertension, commonly observed in individuals with metabolic syndrome, represents a critical component of the complex interplay between cardiovascular and metabolic disorders (6). One of the main factors influencing the initiation and progression of hypertension in the metabolic syndrome is oxidative stress. The impaired redox balance promotes endothelial dysfunction and vascular inflammation, increasing vascular resistance and blood pressure (10). Nitric oxide (NO), a potent vasodilator, plays a crucial role in regulating blood pressure (5). In metabolic syndrome, NO synthesis is dysregulated, partly caused by overexpression of inducible nitric oxide synthase (iNOS), resulting in reduced NO bioavailability (74, 75). Furthermore, alteration in the level of phosphorylation of endothelial nitric oxide synthase (eNOS), especially the ratio of phosphorylated eNOS (P-eNOS) to total eNOS, can also affect NO production and vascular tone (76, 77). Besides, Arginase-2 (Arg2), an enzyme that competes with eNOS for the substrate L-arginine, can decrease NO production and contribute to endothelial dysfunction in metabolic syndrome-associated hypertension (78). The pathophysiology of hypertension has been linked to the dysregulated expression of transcription factors involved in the cellular response to hypoxia, hypoxia-inducible factors (Hif)-1β and Hif-2α (79). These underlying mechanisms, including oxidative stress, NO dysregulation, iNOS, P-eNOS/eNOS imbalance, Arg2, and Hif signaling, collectively contribute to the development and maintenance of hypertension in the context of metabolic syndrome. Understanding these mechanisms may pave the way for novel therapeutic interventions, such as vanillic acid, to target hypertension in individuals with metabolic syndrome. Further research is needed to elucidate the specific roles of these mechanisms and evaluate the potential efficacy of interventions in clinical situations.

*In vivo*

The administration of vanillic acid to hypertensive rats increased NO metabolite concentration in plasma, as well as SOD, catalase (CAT), glutathione peroxidase (GPx), reduced glutathione (GSH), vitamin C, and vitamin E. Moreover, it reduced lipid peroxidation products**,** including conjugated dienes, lipid hydroperoxides, and TBARS. The obtained data disclosed that vanillic acid also decreased systolic and diastolic blood pressures (80). In another study, the supplementation of vanillic acid to hypertensive rats ameliorated left ventricular function and enhanced eNOS expression. It also reduced mean arterial pressure (MAP) and heart rate (81). The administration of vanillic acid to rats with ischemia-reperfusion injury boosted eNOS messenger ribonucleic acid (mRNA) expression levels in cardiac tissue and lowered iNOS mRNA expression. It also attenuated left ventricular end-diastolic pressure. Vanillic acid amended the symptoms of particulate matter 10 (PM10) exposure by increasing the amounts of GPx, CAT, and SOD in blood, decreasing the plasma levels of malondialdehyde (MDA) and xanthine oxidase (XOX), and attenuating blood pressure (82) (Table 4). Furthermore, in rats with cardiotoxicity, vanillic acid could restore heart rate and systolic blood pressure (83). In rats with pulmonary arterial hypertension, vanillic acid administration increased P-eNOS/eNOS levels and NO amounts. It also declined pulmonary tissue fibrosis, protein and gene expression of Arg2, Hif-1β, and Hif-2α, besides right ventricular systolic pressure (84).

Finally, administration of vanillic acid has demonstrated favorable findings in several models of hypertension. The underlying hypotensive effects of vanillic acid have been observed in various studies. Administration of vanillic acid has been shown to increase the concentration of NO metabolites in plasma, associated with vasodilation and improved blood pressure regulation. Vanillic acid supplementation has also been found to enhance anti-oxidant enzyme activity, including SOD, CAT, and GPx, leading to a reduction in oxidative stress and maintenance of vascular health. Additionally, vanillic acid has demonstrated its ability to decrease lipid peroxidation products, indicating its potential to prevent oxidative damage to blood vessels. Furthermore, vanillic acid has been reported to modulate the expression of eNOS, promoting vasodilation and lowering blood pressure. These effects contribute to the hypotensive properties of vanillic acid and suggest its potential as a natural intervention for individuals with hypertension. Further research is necessary to understand the underlying mechanisms fully and explore the clinical implications of vanillic acid in managing high blood pressure. 

## Conclusion

The review highlights some potential benefits of vanillic acid as an intervention for metabolic syndrome and its components, including diabetes, dyslipidemia, obesity, and hypertension, in preclinical studies. Accumulating evidence from *in vitro *and *in vivo* studies supports the therapeutic potential of vanillic acid in managing the complex components of metabolic syndrome. In diabetes, vanillic acid shows favorable properties by activating the AMPK/Sirt1/PGC-1α pathway, modulating insulin signaling pathways such as Akt and ERK1/2, and regulating enzymes and proteins involved in glucose and lipid metabolism. In hyperlipidemia, vanillic acid demonstrates advantageous effects by increasing HDL-C levels, reducing serum cholesterol, triglycerides, FFA, LDL-C, VLDL-C, HMG-CoA reductase activity, and lecithin cholesterol acyl transferase activity. In obesity, vanillic acid acts by inhibiting adipogenesis markers PPARγ and C/EBPα, activating the AMPK signaling pathway, reducing lipid accumulation and inflammation, promoting thermogenesis, enhancing glucose tolerance, and improving insulin resistance. Lastly, in hypertension, vanillic acid exerts hypotensive effects by increasing nitric oxide metabolites, enhancing anti-oxidant enzyme activity (SOD, CAT, GPx), reducing lipid peroxidation, and modulating eNOS expression. These findings suggest vanillic acid as a natural intervention for hypertension. However, further research is required to understand the underlying mechanisms and clinical implications fully. Overall, vanillic acid appears to have efficacy as a potential therapeutic intervention for various components of metabolic syndrome, but further research is needed to determine its effectiveness and safety in humans.

## Data Availability

Data sharing does not apply to this article as no datasets were generated or analyzed during the current study.
